# Practitioner's Bookshelf

**Published:** 1892-01-16

**Authors:** 


### PRACTITIONERS' BOOKSHELF.

### FIRST LINES IN MIDWIFERY.*

This book, which forms one of Cassell's Manual Series, is written for the use of students attending their first cases, and for midwives. The first three chapters are devoted to anatomical and mechanical considerations, which are clearly explained. Rules for the avoidance of hæmorrhage and septic infection should insure, as far as possible, the absence of these accidents. For a last resource in post partum hæmorrhage, a way of compressing the uterus between the two hands is given, which we do not remember to have seen mentioned elsewhere. The two sides of the uterus, he says, can be so pressed together that no bleeding can take place, even if the uterus does not contract. If this really acts as well in practice as in theory, deaths from post partum hæmorrhage can hardly occur. We cannot, however, agree with the author when, in speaking of plugging the vagina for placenta previa, he says, "as a means of checking hæmorrhage it is quite useless." Plugging is a perfectly scientific method of proceeding. The distension of the vagina and the pressure on its nerves acts reflexly on the uterus, causing intense desire to bear down and increased uterine contractions. The latter compress the bleeding vessels, and also press the presenting part against them. Certainly, it should be done while assistance is being sent for. With the thorough antiseptic precautions which he advocates, we are in entire agreement. Whether one in 2,000 corrosive sublimate is too strong or not, is perhaps a matter of opinion. Operation wounds are kept aseptic by a strength of one in 4,000, which we should have thought quite strong enough for obstetric purposes. The frequent use of antiseptic douches in the lying-in period is very properly insisted on. A new and ready method of measuring the antero-posterior diameter of the pelvis without an instrument is clearly explained by diagrams, and should prove very useful. With regard to the revival of the still-born, we are sorry to see the Sylvester method only given; there can be no doubt but that the method of Marshall Hall has many advantages over it. Notwithstanding these criticisms which we have felt it our duty to make, the whole book is free from the common fault of unnecessary condensation, while useless padding is conspicuous by its absence. It will, no doubt, prove very useful to those for whom it is intended.

### SAVILL'S PRESCRIBERS' COMPANION.†

The second edition of Dr. Savill's useful and most convenient little work has just been issued. Whilst carefully conserving many old and well-tried prescriptions, it introduces not a few that have the double merit of being well up to date and thoroughly tried and proved. It is wonderful how much practical information has been condensed and made available in this little shilling book. Beginning with baths and their modes of preparation, it goes on to mixtures, pills, powders, lotions, mouth washes, ointments, nutrient enemata, suppositories, inhalations, and, indeed, includes the whole round of compounds that may be administered in every variety and stage of sickness and disease. Dr. Savill introduces pages on massage, the application of electricity,

* "First Lines in Midwifery." By Dr. G. E. Herman. (Cassell and Co.)

† London: John Bale and Sons.

the artificial feeding of infants, invalid diets, and many other subjects of every-day importance. This Monograph is one for daily use. It is a consultant which can be always at hand. Every busy practitioner, more especially young men and those in extensive general practice, should carry it in the pocket or place it upon the consulting-room table. We know of no other book of the same size and price which contains anything like the same amount of valuable information in so very convenient a form.

---

### LETTS'S MEDICAL DIARY.

"Letts's Medical Diary" is just published, and a copy has been forwarded to us. The little book is all that a medical man wants in the way of a diary. It contains, of course, the usual calendar, registry of births and deaths, abstracts of laws regarding medical practice, and the thousand-and-one small aids, hints, and suggestions which the busy practitioner so constantly needs. In addition, there are posological tables, a nurses' directory, tables of weights and measures, and general *multum in parvo*. The book is beautifully got up, of a convenient size, and suitable for the pocket or for the consulting-room table. We cordially commend it to all our readers.

